# PIK3CA mutation detection in metastatic biliary cancer using cell-free DNA

**DOI:** 10.18632/oncotarget.5432

**Published:** 2015-10-16

**Authors:** Seung Tae Kim, Maruja Lira, Shibing Deng, Sujin Lee, Young Suk Park, Ho Yeong Lim, Won Ki Kang, Mao Mao, Jin Seok Heo, Wooil Kwon, Kee-Taek Jang, Jeeyun Lee, Joon Oh Park

**Affiliations:** ^1^ Division of Hematology-Oncology, Department of Medicine, Samsung Medical Center, Sungkyunkwan University School of Medicine, Seoul, Korea; ^2^ Precision Medicine, Oncology Research Unit, Pfizer, Inc, San Diego, CA, USA; ^3^ WuXi AppTec, Shanghai, China; ^4^ Department of Surgery, Samsung Medical Center, Sungkyunkwan University School of Medicine, Seoul, Korea; ^5^ Department of Pathology, Samsung Medical Center, Sungkyunkwan University School of Medicine, Seoul, Korea; ^6^ Innovative Cancer Medicine Institute, Samsung Medical Center, Seoul, Korea

**Keywords:** cell free DNA (cfDNA), PIK3CA mutation, droplet digital PCR (ddPCR)

## Abstract

*PIK3CA* mutation is considered a good candidate for targeted therapies in cancers, especially biliary tract cancer (BTC). We evaluated the utility of cell free DNA (cfDNA) from serum by using droplet digital PCR (ddPCR) as an alternative source for PIK3CA mutation analysis. To identify matching archival tumour specimens from serum samples of advanced BTC patients, mutation detection using ddPCR with Bio-Rad's PrimePCR mutation and wild type assays were performed for PIK3CA p.E542K, p.E545K, and p.H1047R. Thirty-eight patients with metastatic BTC were enrolled. Only one (BTC 29T) sample (*n* = 38) was positive for PIK3CA p.E542K and another (BTC 27T) for p.H1047R mutation; none was positive for PIK3CA p.E545K. Matched serum sample (BTC 29P) was positive for PIK3CA p.E542K with 28 mutant copies detected, corresponding to 48 copies/ml of serum and an allelic prevalence of 0.3%. Another matched serum sample (BTC 27P) was positive for PIK3CA p.H1047R with 10 mutant copies detected, i.e. 18 copies/ml and an allelic frequency of 0.2%. High correlation was noted in the PIK3CA mutation status between tumour gDNA and serum cfDNA. Low-level PIK3CA mutations were detectable in the serum indicating the utility of cfDNA as a DNA source to detect cancer-derived mutations in metastatic biliary cancers.

## INTRODUCTION

Biliary tract carcinomas (BTCs) are a group of tumours arising from the epithelial cells of intra- and extra-hepatic biliary ducts and gallbladder. They can be divided into gallbladder carcinoma (GBC) and cholangiocarcinoma (CC). BTC is a rare disease with poor prognosis. Its incidence is increasing, accounting for 3% of all gastrointestinal tumours [1], but regional differences are well described [2, 3]. Only 10% of patients present with early stage disease and are considered candidates for curative resection. The prognosis is poor for the majority of patients with metastatic or inoperable BTC with median survival of less than 1 year [4].

Tumour genetics have come to shape the paradigm of personalised medicine among a range of various tumour types. As a result, many molecular techniques have been developed and are in clinical use to molecularly profile tumours and identify potential therapeutic targets. Examples of this include the use of v-erb-b2 erythroblastic leukaemia viral oncogene homolog 2 (HER2) blocking antibodies in breast cancer, epidermal growth factor receptor (EGFR) and anaplastic lymphoma kinase (ALK) inhibitor in lung cancer [5, 6], specific v-raf murine sarcoma viral oncogene homolog B1 (BRAF) inhibitors in melanoma [7], and selection of EGFR blocking antibodies for the treatments of v-Ki-ras Kirsten rat sarcoma viral oncogene homolog (KRAS) wild-type colon cancers [8]. To date, no approaches to identify biomarkers in BTC have been developed. BTCs have a spectrum of mutations in established oncogenes and tumour suppressor genes including *KRAS* [9, 10], *BRAF* [11, 12], *EGFR* [13, 14], *HER2* [15], cyclin-dependent kinase inhibitor 2A (*CDKN2A*) [16], tumour protein p53 (*TP53*) [17], SMAD family member 4 (*SMAD4*) [18, 19], serine/threonine kinase 11 (*STK11*) [20], and the catalytic, alpha subunit of phosphoinositide-3 kinase, (*PIK3CA*) [21]. It is a member of the PI3K signal pathway, which is activated by growth factors such as IGF-1, HGF, and EGF. Binding of the growth factors to the receptor tyrosine kinase leads to proliferation, angiogenesis and cell metabolism [22]. PI3KCA mutation is found in a variety of cancers, considered as a prime drug target and biomarker for anti-cancer therapy [23]. The effect of the mutation status of PIK3CA on anti-EGFR therapies has been studied in KRAS wild type colorectal cancers [24]. Our group also reported the correlation between the mutation status of PIK3CA and the activity of the anti-EGFR therapy, erlotinib, in BTCs [25]. Furthermore, Numerous agents that focus the PI3K pathway are currently examined in various types of tumours [26, 27].

Currently, *PIK3CA* mutations are usually assessed in surgical tissue specimens. However, isolation of sufficient DNA of adequate quality for biomarker analysis from such surgical tissue is not always possible. Moreover, it can be difficult to obtain tumour tissue from patients with metastatic or inoperable BTC. Even in prospectively conducted clinical trials, <50% of patients had tumour tissues available for mutation analysis [28].

Cell free DNA (cfDNA) may be used as a DNA source to detect cancer cell derived mutations [29]. Studies using cfDNA were able to identify the same mutations in the patient's blood as had been identified in the solid tumours for various types of tumours. A significant advantage of the use of cfDNA is that it can be obtained repeatedly and noninvasively from all BTC patients, irrespective of a patients’ characteristics. However, mutant DNA originating from the tumour represents only a small fraction of total cfDNA [29] and therefore is often not detectable using standard PCR.

By using droplet digital PCR (ddPCR), we intended to evaluate the usefulness of circulating tumour DNA from serum as an alternative source for PIK3CA mutation analysis.

## RESULTS

### Patients' characteristics

Thirty-eight recurrent or metastatic BTC patients were enrolled in this analysis. The median age of all patients was 58 years (range, 33 to 72) at study-entry and male/female ratio was 1.9/1.0. Table 1 summarised the patients’ characteristics. The majority of patients had histologically either moderately or poorly differentiated type of biliary adenocarcinoma and 60.5% of patients had more than 2 metastatic lesions.

**Table 1:** Patient characteristics (*N* = 38)

Variables	N	%
Age, years		
Median	58	
Range	33–72	
Sex		
Female	13	34
Male	25	66
Disease type		
Intra-hepatic cholangiocarcinoma	15	39
Extra-hepatic cholangiocarcinoma	9	24
Gall Bladder cancer	14	37
Pathology, adenocarcinoma		
Well differentiated	1	3
Moderate differentiated	21	55
Poorly differentiated	13	34
Well to Moderate	1	3
Moderate to poor	2	5
No. of metastatic site		
1	15	39
2 ≤	23	67

### Analytical sensitivity and specificity

**Figure 1:**
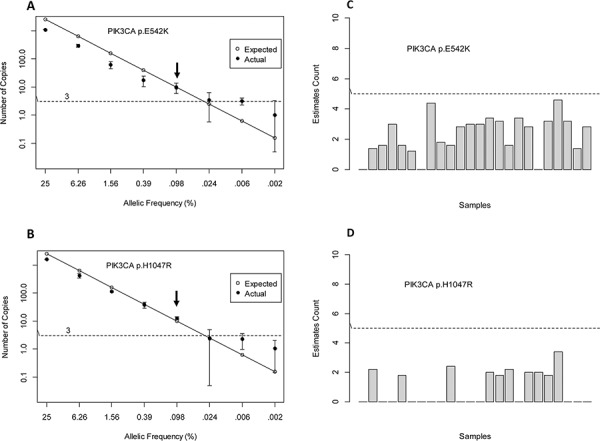
Sensitivity and Specificity

To evaluate linearity and LoD of each assay, we used isogenic reference DNA derived from an engineered mutant cell line of known mutation frequency. DNA containing 50% mutant allele was serially diluted with increasing amounts of isogenic wild type (wt) DNA at the following mutant allele frequencies: 25%, 6.26%, 1.56%, 0.39%, 0.098%, 0.024%, 0.006% and 0% (100% wt). A total of 30 ng of input DNA with varying proportions of mutant to wild type DNA was subjected to droplet digital PCR. All reactions were done in triplicates. Figure 1A and 1B depict analytical linearity and LoD for each assay. Both assays showed linear distribution of mutant alleles as a function of allelic frequencies displaying a wide dynamic range spanning 4 orders of magnitude. Based on confidence interval for Poisson parameter, a sample is positive if the average mutant copies detected is 3 copies and above per reaction.

We determined that the LoD for PIK3CA p.E542K and p.H1047R is at 0.1% mutant allele frequency. This LoD is consistent with the increased variability observed at concentrations below 0.1%. This frequency corresponds to the detection of 10–13 mutant copies/~11,000 wild type copies. A comparison was made between expected mutant copies given 30 ng of input DNA and actual mutant copies. For PIK3CA p.E542K assay, the actual mutant copies detected was lower than expected. This was not observed for PIK3CA p.H1047R assay. To assess assay specificity, we tested genomic DNA obtained from 24 healthy individuals. On average, we observed no false positive counts that were below our threshold of 5 copies/reaction (Figure 1C and 1D).

### The detection of PIK3CA mutation in both tumour tissue and serum

Tumour samples were initially tested for presence of mutations corresponding to PIK3CA p.E542K, p.E545K, and p.H1047R. Of the 38 tumour samples analysed, only two samples were positive for PIK3CA mutations. Tumour samples BTC27 and BTC29 were positive for mutations corresponding to PIK3CA p.H1047R or p.E542K, present at a frequency of 12.4% and 19% respectively (Tables 2 and 3). None of the samples was positive for mutation corresponding to PIK3CA p.E545K (data not shown).

**Table 2:** PIK3CA p.H1047R mutational analysis

PIK3CA p.H1047R				
	Tumor		Serum	
Sample Name	% Mutant Freq.	Mutant (copies/ml)	Wildtype (copies/ml)	% Mutant Freq.
BTC 1	0.0	0	98800	0.0
BTC 2	0.0	0	37920	0.0
BTC 3	0.0	0	19528	0.0
BTC 4	0.0	0	151000	0.0
BTC 5	0.0	0	102667	0.0
BTC 6	0.0	0	47200	0.0
BTC 7	0.0	0	232000	0.0
BTC 8	0.0	0	76233	0.0
BTC 9	0.0	0	23200	0.0
BTC 10	0.0	0	37542	0.0
BTC 11	0.0	0	4552	0.0
BTC 12	0.0	1	9244	0.0
BTC 13	0.0	0	10743	0.0
BTC 14	0.0	0	3000	0.0
BTC 15	0.0	0	4863	0.0
BTC 16	0.0	0	31108	0.0
BTC 17	0.0	0	5685	0.0
BTC 18	0.0	0	4213	0.0
BTC 27	12.4	18	9793	0.2

**Table 3:** PIK3CA p.E542K mutational analysis

PIK3CA p.E542K				
	Tumor		Serum	
Sample Name	% Mutant Freq.	Mutant (copies/ml)	Wildtype(copies/ml)	% Mutant Freq.
BTC 19	0	0	965	0.0
BTC 20	0	0	8203	0.0
BTC 21	0	0	1364	0.0
BTC 22	0	0	12640	0.0
BTC 23	0	0	4640	0.0
BTC 24	0	0	66522	0.0
BTC 25	0	0	16526	0.0
BTC 26	0	0	35051	0.0
BTC 28	0	0	62491	0.0
BTC 29	19	48	15586	0.3
BTC 30	0	0	22519	0.0
BTC 31	0	0	82133	0.0
BTC 32	0	0	65918	0.0
BTC 33	0	0	42862	0.0
BTC 34	0	0	53143	0.0
BTC 35	0	0	39589	0.0
BTC 36	0	0	93333	0.0
BTC 37	0	0	17612	0.0
BTC 38	0	0	77808	0.0

**Figure 2:**
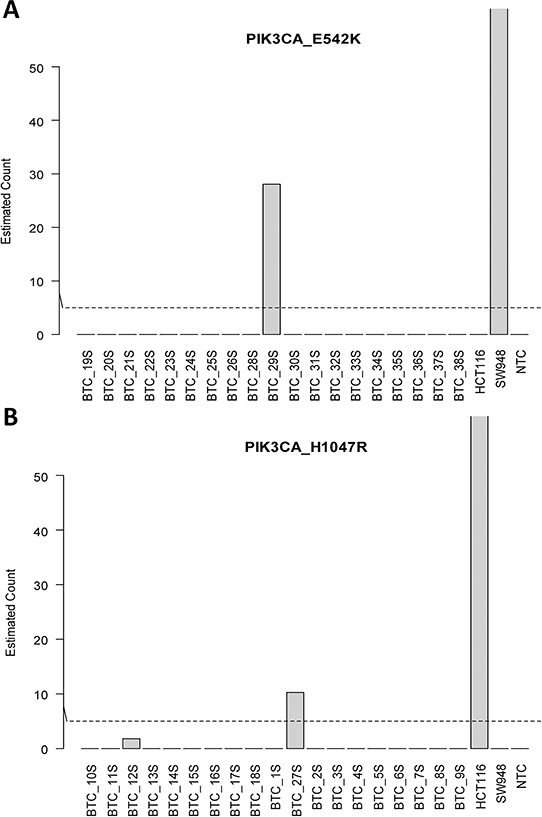
Detection of circulating tumor DNA in serum **A.** PIK3CA_E542K and **B.** PIK3CA_H1047R.

To maximise detection of circulating tumour DNA (ctDNA) from a limited amount of source material (~0.5–1.5 ml of serum), all isolated cfDNA was singly used for genotyping. This necessitated dividing the 38 samples into two groups, one set for each assay. Figure 2 show mutant copies detected in serum. Matched serum sample (BTC 29S) was positive for PIK3CA p.E542K with 28 mutant copies detected (Figure 2A) corresponding to 48 copies /ml of serum and an allelic prevalence of 0.3% (Table 3). For PIK3CA p.H1047R, serum from patient BTC 27P was positive for the mutation with 10 mutant copies detected (Figure 2B). This equates to equivalent to 18 copies/ml and an allelic frequency of 0.2% (Table 2). For both assays, DNA from control cell lines SW948 (PIK3CA p.E542K+) and HCT116 (PIK3CA p.H1047R+) gave the expected genotype calls. Results from both assays were consistent with its matching tumour genotypes (Tables 2 and 3).

## DISCUSSION

In this study, we utilised ddPCR to determine whether circulating tumour DNA can be used as an alternative source for PIK3CA mutation analysis in patients with metastatic biliary cancer. Analytical validation of reference DNA with defined allelic frequencies showed an assay sensitivity of 0.1%. In the germline DNA, no false positive samples were noted that indicated a high degree of specificity. Although the number of positive samples was limited, there was high agreement in PIK3CA mutation status between tumour gDNA and serum cfDNA. Low level PIK3CA mutations were detectable in serum cfDNA at a frequency of 0.2%–0.3%.

The frequency of PIK3CA mutation in BTC has not been known. Based on our analysis, PIK3CA mutation was detected with very low frequency in BTC. For more exact validation of our analysis, we need to test for more prevalent mutation in order to compare with previously published work. However, there have been few studies for evaluating aberrations of specific markers in BTC using cfDNA. These make the comparison among studies be difficult. Thus, first, we focused PIK3CA mutation, known as novel important duggable marker in metastatic BTC, one of medically unmet needs.

The dramatic development over the past decade of genotype directed, anti-cancer therapies has generated considerable interest in noninvasive strategies using cfDNA for cancer genotyping. For KRAS in colon cancer and EGFR in lung cancer previous studies have suggested that cfDNA may be a reasonable alternative to tumour-based genomic testing for determining mutation status [30, 31]. However, there has been a few data on genomic profiling or cfDNA sequencing for BTC. The acquisition of tumour tissue in unresectable/metastatic BTC is especially difficult due to limited role of surgical resection and anatomic location [32]. Hence, cfDNA could be used more effectively in BTC in terms of conducting genotype directed, anti-cancer therapies. To the best of our knowledge, this is the first study to detect PIK3CA mutation in cfDNA from serum in BTC patient cohort. Due to small number of PIK3CA mutation patients in our cohort, its correlation to response to chemotherapy could not be elucidated in this study.

Discriminating ctDNA from normal cfDNA is aided by the fact that tumour DNA is defined by the presence of mutation. Theoretically, all DNA sequencing methodologies that identify somatic variants could be used easily to identify ctDNA if tumour DNA fragments were abundant in the circulation of patients with cancer, However, detection of ctDNA derived from tumours carries substantial challenges, largely because ctDNA often represents a small fraction (<1.0%) of total cfDNA [29, 33, 34]. Thus, standard sequencing approaches that can only detect tumour derived fragments in patients with high levels of ctDNA is not appropriate to be used in general patients with advanced disease. Droplet digital PCR (ddPCR) is one of newly developed methods that allow for enumeration of rare mutant variants in complex mixtures of DNA. In advanced melanoma, ddPCR showed high sensitivity, with the mutation identified in the tumour tissue matching the mutation in the ctDNA fraction. This finding is consistent to our analysis for PIK3CA mutation in BTC.

Currently, there is no standard unit for the reporting of ctDNA genotyping results. Some studies reported results using copies per mL of blood and others have presented blood genotyping results as the percentage of reactions that are mutant [35–38]. Herein, we reported serum genotyping results in both two ways. Our analysis suggested that both may be useful for reporting of ctDNA genotyping. However, definite comparison between the two ways was impossible because of the sample size. Our sample size was too small to allow us to draw any significant conclusions. Unlike other solid tumors, BTCs have hurdles for biomarker-studies with tumors as well as blood. This tissue availability was a potential limitation of the biomarker analysis in BTC. Moreover, the rarity and short survival of BTC hinders clinicians from conducting definitive trials and from producing rigorous scientific data. Nevertheless, we tried to evaluate the utility of cfDNA as an alternative source for PIK3CA mutation analysis through comparing serum sample with matching archival tumor specimens in BTC. Further, coordination of trials among institutions and cooperative groups, both nationally and internationally, will be the key to improving research-outcomes in BTC. Research and validation for more precise strategies for presentation of blood genotyping results is also needed. These steps will enable physicians to choose molecular targeted agents based on molecular profiling using cfDNA of patients. Dr Turner presented the clinical trial using cfDNA in metastatic breast cancer at 2015 ASCO meeting. Currently, we are also testing the role of cfDNA in refractory cancer patients on progression-free survival in the NEXT-2 trial (NCT#02140463).

In conclusion, blood may be useful alternative source for PIK3CA mutation analysis in BTC. Additionally, ddPCR is a reliable method to detect ctDNA of small fraction from total cfDNA in blood. To acquire tumour tissue in BTC is more difficult than other cancer types. This hurdle has interrupted the application of personalised medicine using tumour genetics in BTC. Mutational analysis using ddPCR for blood is likely to be helpful for giving BTC patients a drug tailored to the genetic make-up of their tumour.

## MATERIALS AND METHODS

### Patient samples/DNA isolation

Patients with recurrent or metastatic biliary tract cancer (BTC) were enrolled in this study. BTCs included intrahepatic or extrahepatic cholangiocarcinoma (CC) and gall bladder (GB) cancer. For each patient, blood samples with matching archival biliary tumour specimens were obtained from Samsung Medical Center (Seoul, Korea) with prior patient's informed consent and with approvals from Samsung Medical Center's ethical committee/internal review board. DNA was isolated from two 10 μm slide sections of formalin-fixed paraffin-embedded (FFPE) tissue using QIAamp DNA FFPE Tissue kit (Qiagen, Valencia, CA, USA) and DNA concentrations were determined using Qubit 2.0 (Life Technologies, Carlsbad, USA). A pathologist reviewed each slide and verified the presence of adequate tumour tissue containing more than 50% malignant cells. All experimental procedures were carried out in accordance with guidelines approved by Samsung Medical Center.

### Blood samples and circulating cell free DNA (cfDNA) isolation and quantification

For each enrolled patient, blood was collected during routine blood sampling. Blood samples were immediately processed upon receipt. Serum was isolated from EDTA tubes by centrifugation at 1,600 × *g* for 10 min at 4°C. Serum was aliquoted and stored at −70°C. cfDNA was isolated from 0.5 to 1.8 ml of serum using QiaAmp circulating nucleic acid isolation kit (Qiagen, Valencia, CA, USA) and quantitated using Qubit 2.0 high sensitivity DNA kit (ThermoFisher Scientific, Carlsbad, CA, USA).

### PIK3CA assays/digital PCR analysis

Mutation detection was performed using droplet digital PCR on a QX100™ droplet digital PCR system (Bio-Rad, Hercules, CA, USA) using Bio-Rad's PrimePCR mutation and wild type assays for PIK3CA p.E542K, p.E545K, and p.H1047R. The TaqMan-based probes corresponding to mutant or wild type alleles were labelled with either 6-FAM or HEX fluorophores, respectively. DNA from 24 healthy individuals was obtained from Coriell Cell Repositories (Camden, NJ, USA). Control cell lines SW948 (PIK3CA E542K+), HCT15 (PIK3CA E545K+), and HCT116 (PIK3CA H1047R+) were purchased from the American-Type Culture Collection (Manassas, VA, USA). Cell line DNA was isolated using QiaAmp DNA isolation kit (Qiagen, Valencia, CA, USA) and concentration determined using Qubit 2.0.

Isogenic reference DNA of known mutant allele frequency was used (Horizon Diagnostics, Cambridge, UK) to assess each assay's limit of detection. Thirty nanograms of DNA containing serially diluted mutant DNA were combined with a solution containing 1× ddPCR supermix for probes (no dUTP), 1× mutant (FAM-labelled) and wild type (HEX-labelled) PrimePCR ddPCR assays, and 3 units of *Mse*I restriction enzyme (New England Biolabs Inc., Ipswich, MA, USA) in a final volume of 20 μl. All reactions were performed in triplicates. The mixture was compartmentalised into approximately 20,000 oil droplets using a QX100 droplet generator. Emulsified PCR mix was transferred to a 96-well plate and PCR-amplified on a S1000™ thermal cycler (Bio-Rad, Hercules, CA, USA) under the following conditions: 95°C for 10 min followed by 40 cycles of 94°C for 30 s and 55.8°C for 1 min followed by 10 min incubation at 98°C and held at 4°C. The optimal annealing temperature for each assay was found to be 55.8°C by using temperature gradient analysis (55°C–65°C) as it led to the maximum signal differences between positive and negative droplets. After amplification, the PCR plate was transferred to a droplet reader where droplets were streamed single file past an optical detector and counted. Data analysis was performed using QuantaSoft v1.2.10.0 software where target concentration was calculated as copies/reaction.

For serum genotyping, cfDNA eluate isolated from 0.5 ml–1.5 ml of serum was concentrated to a volume of 10 μl and subjected to ddPCR as described above. The number of mutant copies was converted to reflect copies/ml of serum used.

### Statistical analysis

The copy number of target DNA (*x*) in a droplet is typically assumed to follow a Poisson distribution

$$P\left(x=k\right)=\lambda^{k}e^{-\lambda }/k$$ (1)

where λ is the expected number of copies in the droplet. Under this assumption, the probability to have a negative droplet (copy number = 0) is

$$P\left(x=0\right)=e^{-\lambda }.$$ (2)

By counting the number of positive (or negative) droplets, we can estimate λ by

$$\hat{\lambda }=-\ln\left(1-\frac{T}{N}\right),$$ (3)

where *T* is the total number of positive droplets detected among *N* droplets, and has a binomial distribution with the proportion parameter *p* estimated by

$$\hat{p}=T/N$$ (4)

The total copy number of targeted DNA in the sample is Nλ. We define the limit of detection (LoD) of a targeted DNA as $k_{LoD}$, such that we have 95% confidence and at least one copy of the target DNA is detected in the sample. That is, the lower 95% confidence interval of Nλ has to be greater than or equal to 1.

$${\text{Nλ}}_{\text{L}}=-\text{N}\cdot \ln(1-p_{\text{L}})\geq 1$$ (5)

where the subscript *L* indicates lower confidence limit. To find $p_{L}$, the lower confidence limit of binomial parameter p, we use Clopper-Pearson exact method [39] instead of the more commonly used method based on normal approximation, as the copy number is typically very small.

$${\hat{p}}_{L}={\left(1+\frac{N-k+1}{kF(0.025,2k,2(N-k+1))}\right)}^{-1}$$ (6)

where F() is the F distribution quantile function. The limit of detection $k_{LoD}$ is the smallest k such that the lower confidence limit

$$-N\cdot ln(1-{\hat{p}}_{L})\geq 1.$$ (7)

For ddPCR with a typical N~10000–20000, we have $k_{LoD}\;=4.$ Considering there are three target DNAs, we set a more stringent α level at 0.05/3 to control the inflated Type 1 error from multiple testing, and this leads to the final detection threshold $k_{LoD}\;=5$. When technical replicates exist, we define the threshold using the average copy number such that the average lower confidence limit is at least one. The resulted threshold for the average copy number is 2.77 for three replicates, and we round it up to 3.
